# Anaerobic methane oxidation coupled to manganese reduction by members of the *Methanoperedenaceae*

**DOI:** 10.1038/s41396-020-0590-x

**Published:** 2020-01-27

**Authors:** Andy O. Leu, Chen Cai, Simon J. McIlroy, Gordon Southam, Victoria J. Orphan, Zhiguo Yuan, Shihu Hu, Gene W. Tyson

**Affiliations:** 1 Australian Centre for Ecogenomics, School of Chemistry and Molecular Biosciences, The University of Queensland, Brisbane, QLD Australia; 2 Advanced Water Management Centre, Faculty of Engineering, Architecture and Information Technology, The University of Queensland, Brisbane, QLD Australia; 3 School of Earth & Environmental Sciences, The University of Queensland, Brisbane, QLD 4072 Australia; 4 Department of Geological and Planetary Sciences, California Institute of Technology, Pasadena, CA 91106 USA

**Keywords:** Biogeochemistry, Biogeochemistry, Environmental microbiology

## Abstract

Anaerobic oxidation of methane (AOM) is a major biological process that reduces global methane emission to the atmosphere. Anaerobic methanotrophic archaea (ANME) mediate this process through the coupling of methane oxidation to different electron acceptors, or in concert with a syntrophic bacterial partner. Recently, ANME belonging to the archaeal family *Methanoperedenaceae* (formerly known as ANME-2d) were shown to be capable of AOM coupled to nitrate and iron reduction. Here, a freshwater sediment bioreactor fed with methane and Mn(IV) oxides (birnessite) resulted in a microbial community dominated by two novel members of the *Methanoperedenaceae*, with biochemical profiling of the system demonstrating Mn(IV)-dependent AOM. Genomic and transcriptomic analyses revealed the expression of key genes involved in methane oxidation and several shared multiheme *c*-type cytochromes (MHCs) that were differentially expressed, indicating the likely use of different extracellular electron transfer pathways. We propose the names “*Candidatus* Methanoperedens manganicus” and “*Candidatus* Methanoperedens manganireducens” for the two newly described *Methanoperedenaceae* species. This study demonstrates the ability of members of the *Methanoperedenaceae* to couple AOM to the reduction of Mn(IV) oxides, which suggests their potential role in linking methane and manganese cycling in the environment.

## Etymology

“*Candidatus* Methanoperedens manganicus” sp. nov

“*Candidatus* Methanoperedens manganireducens” sp. nov

“*Candidatus* Methanoperedens manganicus”: man.ga’ni.cus. N.L. neut. n. manganum, manganese; L. suff. -icus, belonging to, pertaining to; N.L. masc. adj. manganicus, pertaining to manganese.

“*Candidatus* Methanoperedens manganireducens”: man.ga.ni.re.du’cens. N.L. neut. n. manganum, manganese; L. pres. part. reducens, bringing back, restoring; N.L. part. adj. manganireducens, reducing manganese compounds.

## Introduction

Methane is a greenhouse gas that is ~28 times more potent than carbon dioxide [1], and its fate in environmental systems has important implications for Earth’s climate. Anaerobic oxidation of methane (AOM) is a globally important microbiological process that prevents the atmospheric release of a substantial proportion of the methane from natural sediments (>85% in some environments) [2–4]. Several archaeal lineages within the class Methanomicrobia have been shown to mediate AOM, including the ANME-1, ANME-2a-c, *Methanoperedenaceae* (formerly known as ANME-2d), and the ANME-3. In the absence of a pure culture, “omic” and single cell visualization approaches have demonstrated the ability of different anaerobic methanotrophic archaea (ANME) lineages to oxidize methane coupled to the reduction of sulfate, in concert with a syntrophic partner (ANME-1, 2a–c, 3) [5–8], and nitrate (*Methanoperedenaceae*) [9].

In addition to these electron acceptors, several environmental studies have provided evidence for the potential for AOM coupled to the reduction of iron (Fe(III)) and manganese (Mn(IV)) in marine and freshwater environments based on geochemical measurements [10–15]. Given the large amounts of iron (~730 Tg/yr) and manganese (19 Tg/yr) being deposited into continental margins [16, 17], and that these metals can be oxidized and reduced up to 300 times before burial [18], AOM coupled to metal reduction could represent an important global methane sink [15].

The ability of microorganisms to mediate AOM coupled to the reduction of iron and manganese oxides was first demonstrated in incubation experiments with marine sediments [15]. Subsequent analyses of these sediment incubations, using fluorescence in situ hybridization coupled to secondary ion mass spectrometry (FISH-SIMS), identified archaeal populations that were morphologically similar to ANME-2 as being active and likely responsible for the observed Mn-driven AOM [19]. Similar FISH-SIMS studies have also demonstrated the ability of marine sediment ANME-2 populations to couple AOM to iron reduction [6]. In addition, freshwater sediment bioreactors dominated by a member of the genus “*Ca*. Methanoperedens sp. MPEBLZ” (within the family *Methanoperedenaceae*) were shown to exhibit AOM activity during short term incubations (3 days) when nitrate was substituted for either Fe(III) or Mn(IV) oxides [20]. More recently, “*Ca*. Methanoperedens ferrireducens” was enriched in a long-term culture shown to couple AOM to Fe(III) reduction [21]. Based on *meta*-omic analysis, “*Ca*. M. ferrireducens” was hypothesized to oxidize methane using a unique set of multiheme cytochromes (MHCs) for extracellular dissimilatory Fe(III) reduction [21].

Despite preliminary evidence for AOM coupled to Mn(IV) reduction [15, 20], long-term performance and mass balance data, combined with a detailed understanding of the microbial community and pathways responsible for this metabolism are still lacking. To assess the potential for Mn(IV)-dependent AOM, a bioreactor fed with methane and Mn(IV) oxides in the form of birnessite was operated for 480 days. Bioreactor performance data and *meta*-omic analysis was used to identify two novel members of the *Methanoperedenaceae* capable of AOM coupled to Mn(IV) reduction and the likely metabolic strategies they employ to perform this metabolism.

## Materials and methods

### Birnessite synthesis

Birnessite was synthesized at ambient pressure and temperature by reducing KMnO_4_ with C_3_H_5_NaO_3_ [22]. Specifically, 5 ml of 60% C_3_H_5_NaO_3_ was mixed with 500 ml of a 1 g l^−1^ KMnO_4_ solution for 2 h and the precipitate was harvested by centrifugation. The product was washed five times with Milli-Q water, and then freeze-dried and stored (for up to 4 months) in a drying cabinet at room temperature before use.

### Bioreactor setup and operation

**Fig. 1:**
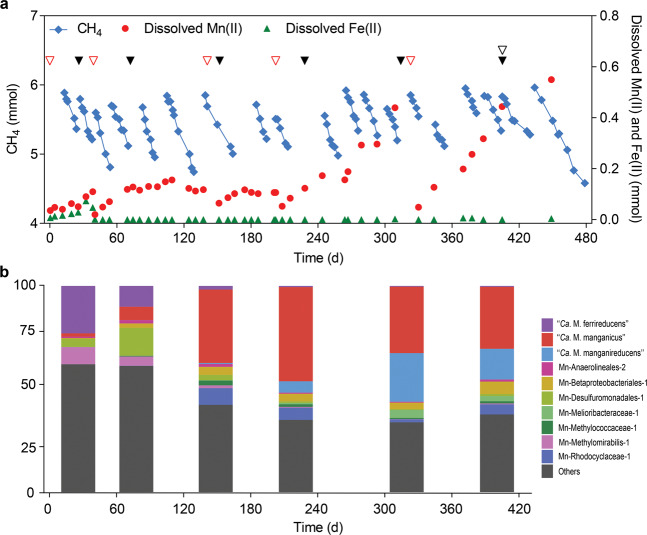
Monitoring of bioreactor performance and community composition. **a** Bioreactor profiles for methane, dissolved Mn(II), and dissolved Fe(II) over the course of operation. Note that an increase in dissolved metal species indicates metal oxide reduction but is not a reliable measure of the total rate of reduction due to their adsorption and the formation of Fe/Mn-carbonates. The triangles indicate the addition of birnessite (red) and the sampling for metagenomic (black) and metatranscriptomic analyses (white). **b** Community composition profiles showing the top ten most abundant MAGs over time. The abundance values are given in Supplementary Table 5.

The Mn(IV)-AOM bioreactor was seeded with 200 ml of an enrichment culture dominated by “*Ca*. M. ferrireducens” taken from a parent bioreactor performing Fe(III)-dependent AOM [21] which had been operating for 518 days. The parent bioreactor was initially seeded with organic-rich freshwater sediment from the Gold Creek Reservoir in Brisbane, Australia (27°27′37″ S, 152°52′53″ E) [23], and was fed methane and ferrihydrite [21]. The inoculum was anaerobically transferred into an 830 ml bioreactor and mixed with 500 ml medium, resulting in a working volume of 700 ml and a 130 ml headspace. The medium composition was as described previously [21] and included KH_2_PO_4_ (0.075 g l^−1^), MgCl_2_·7H_2_O (0.165 g l^−1^), CaCl_2_·2H_2_O (0.3 g l^−1^), alkaline trace element solution (0.2 ml l^−1^), and acidic trace element solution (0.5 ml l^−1^). The composition of the alkaline and acidic trace element solutions was described previously [24]. The bioreactor was continuously mixed using a magnetic stirrer at 300 rpm and operated at 22 ± 2 °C. The methane partial pressure in the bioreactor headspace was maintained between 0.6 and 1.2 atm through supplying the bioreactor with a mixed gas comprising 90% CH_4_, 5% CO_2_, and 5% N_2_. Birnessite was pulse fed to the bioreactor as an electron acceptor for AOM (~1–2 g dry weight every 2–4 months; Fig. 1). Every 1–3 months, the stirring of the bioreactor was stopped for 24 h to allow the biomass to settle and ~50 ml of the supernatant was replaced with fresh medium. Nitrogen gas was sparged through the bioreactor headspace during medium replacement to avoid oxygen contamination. The bioreactor was equipped with a pH meter (Oakton, USA) and the pH maintained between 6.8 and 7.5 by manually dosing of a 1 M HCl solution.

Gas samples were taken 2–3 times per week for methane measurement. Overall, 1.2 ml liquid samples were taken 2–4 times per month and filtered through sterile 0.22 μm polyethersulfone filters for the measurement of dissolved Mn(II) and Fe(II). Nitrate, nitrite, sulfate, and sulfide were also measured during reactor operation.

**Fig. 2:**
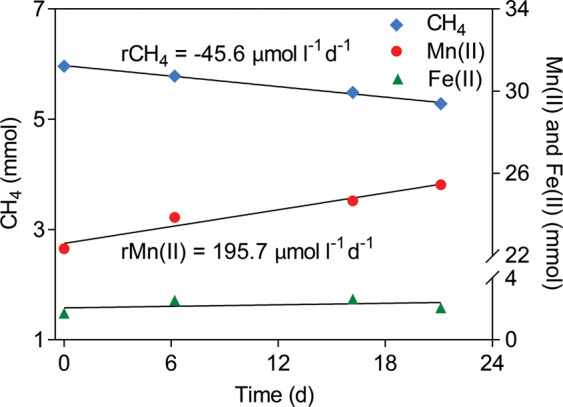
Detailed bioreactor performance measurements for methane oxidation and Mn(II) reduction starting on day 434. Each point for dissolved Mn(II)/Fe(II) represents the average of two measurements. Replication of these analyses is provided in Supplementary Fig. 1. The measured fractions of total Mn(II) are given in Supplementary Table 1.

Intensive testing was conducted on the bioreactor to determine the rates of methane oxidation and Mn(IV) reduction for ~20 day periods starting on days 434 and 456. Methane and extractable Mn(II) (adsorbed Mn(II) and Mn(II) carbonates) were both measured weekly during these periods. Dissolved Mn(II) was only measured at the beginning and end of each test to minimize the loss of Mn(II) in the solid phase due to sampling. Given dissolved Mn(II) represented <3% of the total Mn(II) (Supplementary Table 1) it was not included in the subsequent calculations (Fig. 2). Fe(II) in the liquid and solid phases was also quantified to evaluate the potential for Fe(II) production.

### Free energy calculation

The free energy of Eq. (2) was calculated based on the in situ conditions at the beginning of the intensive bioreactor performance analyses on day 434 using the following concentrations: CH_4_ = 1.37 mM, MnO_2_ = 8.27 mM, H^+^ = 10^−4^ mM, HCO_3_^−^ = 0.116 mM, Mn^2+^ = 0.71 mM. The values of CH_4_ and HCO_3_^−^ were calculated using Henry’s law. The concentration of MnO_2_ was based on its estimated consumption. The value of H^+^ concentration was calculated from the average pH during the incubation. The free energy was calculated at 22 °C.

### Chemical analyses

Filtered bioreactor samples were obtained for the measurement of dissolved Fe(II) and Mn(II). Mn(II) and Fe(II) in the solid phase were extracted as described previously [25–27]. Briefly, the samples were centrifuged at 3267 × *g* for 10 min and the supernatants were discarded. The remaining solids were then treated with (1) 1 M MgCl_2_ at pH 7 for 2 h to extract Mn(II)/Fe(II) in the form of adsorbed Mn(II)/Fe(II) and (2) 1 M sodium acetate at pH 4.5 for 24 h to extract Mn(II)/Fe(II) in the form of Mn(II)/Fe(II) carbonate. Dissolved and extracted Mn(II) and Fe(II) fractions were measured using an Optima 7300DV Inductive Coupled Plasma Optical Emission Spectrophotometer (PerkinElmer, USA). Gaseous methane was measured using a GC-8A Gas Chromatograph (Shimadzu, Japan) equipped with a Porapak Q column and a thermal conductivity detector as described previously [28]. The total methane concentration, including the methane dissolved in the liquid phase, was calculated from its concentration in the gaseous phase using Henry’s Law. Nitrate and nitrite were measured using a Lachat QuickChem8000 Flow Injection Analyzer (Lachat Instrument, USA). Sulfate was measured using an ICS-2000 Ion Chromatograph with an AD25 absorbance and a DS6 heated conductivity detector (Dionex, USA).

Biomass samples (2 ml) were taken on day 26, 72, 152, 228, and 314 for genomic DNA extraction using a FastDNA SPIN kit for soil (MP Biomedicals, USA) following the manufacturer’s protocol. An additional biomass sample was taken on day 405 and stored in Lifeguard Soil Preservation Solution (MoBio, USA) for the subsequent co-extraction of DNA and RNA. The preserved biomass was centrifuged and the sediment (~200 mg) transferred into a 2 ml Lysing Matrix E tube (MP Biomedicals, USA) containing 1.2 ml of lysis solution (750 mM sodium phosphate buffer, pH 7; 500 mM NaCl; 0.5% cethyltrimethyl ammonium bromide; 50 mM EDTA). The sample was homogenized twice using a PowerLyzer Homogenizer (MoBio, USA) for 30 s at 4500 rpm at room temperature (25 °C) with the tube cooled on ice for 60 s in between homogenization steps. Cell debris and solid material were pelleted by centrifugation at 16,000 × *g* for 5 min at 10 °C. The supernatant was subjected to phenol/chloroform/isoamyl alcohol (25:24:1) extraction (pH 6.5), followed by two chloroform extractions. The aqueous phase was divided equally and DNA and RNA purified from each fraction using the DNA Clean & Concentrator-10 Kit (Zymo Research, USA) and the RNeasy MinElute Cleanup Kit (Qiagen, Germany), respectively. Contaminating genomic DNA was removed from the RNA fraction using the Turbo DNA-Free Kit (Thermo Fisher Scientific, USA), and the sample further purified using an RNA Clean & Concentrator-5 Kit (Zymo Research, USA) following the manufacturer’s protocol.

### DNA and RNA library preparation and sequencing

Paired-end libraries were prepared from all bioreactor DNA extracts using the Nextera XT DNA Library Preparation Kit (Illumina, USA). A metatranscriptome was prepared from the RNA extract from day 405. Depletion of rRNA from the extract was performed using the Ribo-Zero Magnetic Kit (Epicentre, USA) as per the manufacturer’s protocol. The RNA was prepared for sequencing using the ScriptSeq Stranded mRNA Library Prep Kit (Illumina, USA) following the manufacturer’s protocol. Both DNA and mRNA libraries were sequenced on a NextSeq 500 (Illumina, USA) platform generating 2 × 150 bp paired-end reads with an average insert length of 300 bp.

### Metagenomic community profiling

GraftM v0.11.1 [29] was used to determine community abundance profiles from the raw metagenomic datasets using a 16S rRNA GraftM package created from Greengenes (release 13_8; 97% OTU clustering) [30].

### Quality control, assembly, and binning of metagenomes

Metagenome reads from day 152, 228, 314, and 405 were trimmed for quality using PEAT and assembled independently using metaSPAdes v3.9.0 both with default parameters. Mapping of quality reads was performed using BamM v1.7.3 with default parameters (https://github.com/Ecogenomics/BamM). Several metagenome assembled genomes (MAGs) were recovered from the assembled metagenomes using uniteM v0.0.14 (https://github.com/dparks1134/UniteM). Redundant MAGs were dereplicated using dRep v1.0.0 using the dereplicate_wf, and the best bins were chosen based on genome completeness. The “*Ca*. Methanoperedens” MAGs were further refined by reassembling the mapped quality trimmed reads with SPAdes using the—careful and—trusted-contigs setting. The “roundup” mode of FinishM v0.0.7 (https://github.com/wwood/finishm) was implemented for additional scaffolding and to resolve ambiguous bases of the “*Ca*. Methanoperedens” MAGs. Completeness and contamination of the population bins were assessed using CheckM v1.0.11 [31] with the “lineage wf” command.

### Functional annotation

For all MAGs, open reading frames (ORFs) were called and annotated using Prokka v.1.12 [32]. Additional annotation was performed using the diamond blastp “verysensitive” setting in Diamond v0.9.18 (https://github.com/bbuchfink/diamond.git) against UniRef100 (accessed March 2017) [33], clusters of orthologous groups (COG) [34], Pfam 31 [35], and TIGRfam (Release: January 2014) [36]. ORFs were also diamond blastp searched against Uniref100 (accessed March 2017) containing proteins with KO ID. The top hit for each gene with an *e*-value < 1e^−3^ was mapped to the KO database [37] using the Uniprot ID mapping files. KO annotations linked to microbial metabolism of interest are summarized with TPM (transcripts per million) values (Supplementary Dataset 1). ORFs from the “*Ca*. Methanoperedens” MAGs were also annotated using the archaeal clusters of orthologous genes (arCOG) (ftp://ftp.ncbi.nih.gov/pub/wolf/COGs/arCOG/) [38]. Finally, genes of interest were further verified using NCBI’s conserved domain search to identify conserved motif(s) present within the gene [39]. Putative multiheme *c*-type cytochromes (MHCs) in the draft MAGs and the metagenome assemblies were identified by searching ORFs for ≥3 CXXCH motifs and cytochrome *c*-type protein domains using hmmsearch (HMMER v.3.1) [40] with PfamA [41]. MHCs with signal peptides identified by SignalP v5.0 [42] and/or predicted to be extracellular by PsortB v3.0 [43] were considered to be relevant to extracellular electron transport.

### Construction of genome trees and taxonomy

The archaeal and bacterial genome trees were constructed using the Genome Taxonomy Database (GTDB v2.2.1, https://github.com/Ecogenomics/GTDBNCBI) with a concatenated set of 122 archaeal-specific and 120 bacterial-specific conserved marker genes (see Supplementary Dataset 2) inferred from genomes available in NCBI (NCBI RefSeq release 83) [44]. These marker genes were identified and aligned in each genome using HMMER v.3.1 [40], concatenated, and trees were constructed using FastTree V.2.1.8 [45] with the WAG + GAMMA models. Support values were determined using 100 nonparametric bootstrapping with GenomeTreeTK. The trees were visualized using ARB [46] and formatted using Adobe illustrator (Adobe, USA). Classification of the bacterial and archaeal genomes were assigned using GTDB-Tk v.0.0.7 using the classify_wf command (https://github.com/Ecogenomics/GtdbTk).

### Comparative genomic analyses

The “*Ca*. Methanoperedens” MAGs identified in this study were compared with publicly available *Methanoperedenaceae* genomes. Average amino acid identity (AAI) between the genomes was calculated using orthologous genes identified through reciprocal best BLAST hits using compareM v0.0.5 (https://github.com/dparks1134/CompareM). Homologous proteins across all available *Methanoperedenaceae* MAGs were identified with OrthoFinder v2.3.3 using default parameters. Gene counts of orthologous groups containing MHCs were used as input for the pheatmap package in R and hierarchical clustering was performed using ward.D2 [47].

### Metatranscriptomic analysis

The metatranscriptomic paired-end reads were quality trimmed using PEAT with default settings and mapped to the dereplicated genome set using BamM v1.7.3. The resulting mapping files were filtered using 0.97 and 0.95 as the minimum percentage values for identity alignment for a mapped read, respectively. DetectM v0.0.3 (https://github.com/geronimp/detectM) was used to determine the counts for the unambiguously mapped mRNA reads for each ORF and to calculate the RNA-TPM based on Eq. (1):

1 $${\rm{TPM}}\,=\,\frac{{\rm{rg}}\,\times\,{\rm{rl}}\,\times\,10^{6}}{{\rm{flg}}\,\times\,{{\rm{T}}}},$$

where rg = reads mapped to gene g, rl = read length, flg = feature length (or CDS length), and *T* = sum of rg × rl/flg for all genes.

## Results and discussion

### Establishment and long-term performance of a Mn-dependent AOM bioreactor

Biomass collected from a bioreactor performing Fe(III)-dependent AOM [21], originally seeded from freshwater reservoir sediment, was used to inoculate a newly established bioreactor supplied with methane, and birnessite as the electron acceptor. An increase in dissolved Mn(II) was observed within the first 50 days, indicating manganese reduction. A relatively small increase in dissolved Fe(II) was observed in the first 50 days, indicating the reduction of residual Fe(III) during that period. From day 140 to 480, methane consumption concomitant with the increase of dissolved Mn(II) (Fig. 1a) was observed with an average rate of methane oxidation of 56.2 μmol l^−^^1^ d^−1^, which is comparable with the average of 62.9 μmol l^−1^ d^−1^ measured in the parent Fe(III)-driven AOM system (calculated from day 200 to 1100; see Supplementary Fig. 4 of Cai et al. [21]). Birnessite was pulse fed to the bioreactor periodically. A dramatic decrease in dissolved Mn(II) (Fig. 1a) after feeding was likely due to it being adsorbed to the added birnessite, as many amorphous oxides have been shown as strong adsorbents for metal ions [48, 49].

To assess the stoichiometry of Mn(IV)-dependent AOM in the bioreactor, Mn(II) production and methane consumption rates were measured during two intensive analyses periods starting on days 434 and 456, respectively (Fig. 2; Supplementary Fig. 1). During these periods, the average methane consumption rate was 44.5 μmol l^−1^ d^−1^ and the average Mn(II) production rate was 184.7 μmol l^−1^ d^−1^. The ratio between the Mn(II) production rate and the methane consumption rate in each analysis period (4.3, days 434–455; 4.0 days 456–479) was close to the calculated stoichiometric ratio of 4:1 (Eq. (2)), demonstrating that AOM was most likely coupled to Mn(IV) reduction in the bioreactor. Based on the in situ conditions of the reactor, AOM coupled to birnessite (simplified as MnO_2_ in Eq. (2)) was estimated to yield a potential free energy of ΔG = −383 kJ mol^−1^ CH_4_.

2 $${\rm{CH}}_{4\left({\rm{aq}}\right) +}4{\rm{MnO}}_{2({\rm{s}})} + 7{\rm{H}}^ + \to {\rm{HCO}}_{3({\rm{aq}})}^ - + 4{\rm{Mn}}^{2 + } + 5{\rm{H}}_2{\rm{O}}.$$

The potential contribution of other electron acceptors to AOM appears to be negligible. Nitrate and nitrite were consistently low (<0.01 mmol), and residual sulfate introduced with the inoculum remained stable in the bioreactor over the course of operation (Supplementary Fig. 2). Substantial Fe(III)-driven AOM is also unlikely given the main product would be unreactive Fe(II)-carbonates (FeCO_3_; accounting for >95% of the Fe(II) produced in the parent bioreactor [21]) and total Fe(II) remained stable during the mass balance analyses periods (Fig. 2). These data collectively support Mn(IV)-dependent AOM as the primary process in the bioreactor.

### Genome recovery and community structure

**Fig. 3:**
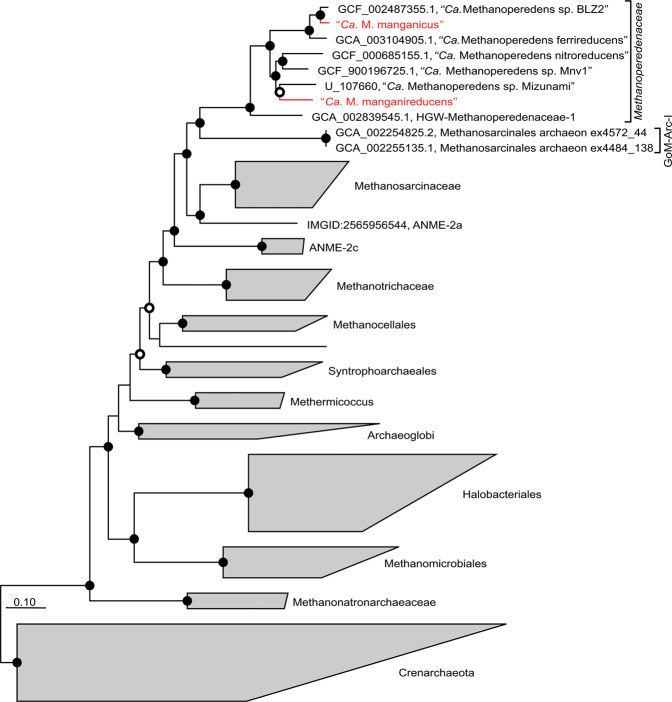
Phylogenetic placement of the dominant “Ca. Methanoperedens” populations. Genome tree showing the phylogenetic placement of the two “*Ca*. Methanoperedens” genomes. The genome tree was inferred using the maximum-likelihood method with a concatenated set of 122 archaeal-specific marker genes, and bootstrap values were calculated using nonparametric bootstrapping with 100 replicates. The “*Ca*. Methanoperedens” genomes from this study are highlighted in red. Black and white dots indicate ≥90% and ≥70% bootstrap values, respectively. The scale bars represent amino acid substitutions per site.

To obtain genomes representing the abundant members of the bioreactor community, metagenomic sequencing was performed on biomass samples from days 26, 72, 152, 228, 314, and 405. A nonredundant set of 21 high-quality MAGs (>77% complete and <5% contamination, based on CheckM) (Supplementary Table 2) was recovered from the metagenomes. The taxonomic affiliation of these MAGs was assessed with archaeal and bacterial genome trees constructed using 122 and 120 conserved single-copy marker genes, respectively (Fig. 3; Supplementary Fig. 3). Taxonomic affiliation of the MAGs was also assessed using GTDB-Tk, which classifies genomes based on their placement in a reference genome tree, relative evolutionary distance, and FastANI distance. This classification was consistent with the genome trees (Supplementary Table 3). Two MAGs (>99% complete and <5% contamination) belonging to the genus “*Ca*. Methanoperedens” were identified and the names “*Candidatus* Methanoperedens manganicus” and “*Candidatus* Methanoperedens manganireducens” are proposed based on their apparent ability to utilize manganese as an electron acceptor. Based on the archaeal genome tree, these MAGs were phylogenetically distinct from “*Ca*. M. ferrireducens” and “*Ca* M. nitroreducens” [9, 21], with “*Ca*. M. manganicus” forming a sub-cluster with “*Ca*. M. nitroreducens sp. BLZ2” [50] and “*Ca*. M. ferrireducens” [21], and “*Ca*. M. manganireducens” being more closely associated with the Mizunami Methanoperedens species [51] (Fig. 3). The average AAI of “*Ca*. M. manganicus” and “*Ca*. M. manganireducens” when compared with their closest sequenced relative “*Ca*. M. nitroreducens sp. BLZ2” (89.2% similar) and “*Ca*. M. nitroreducens sp. IPS” (76.0% similar), respectively, indicates that both of these MAGs represent novel species within the genus “*Ca*. Methanoperedens” (Supplementary Table 4) [52]. “*Ca*. M. manganicus” and “*Ca*. M. manganireducens” have an AAI of 73.3%.

To examine the relative abundances of the MAGs over the course of reactor operation, metagenome reads from each sampling time point were mapped onto the 21 dereplicated MAGs, as well as all publicly available *Methanoperedenaceae* genomes (Supplementary Table 5). Approximately 70% of the reads from each metagenome were mapped onto this genome set for the metagenome samples taken around the time of the metatranscriptomic and mass balance analyses. A large increase in relative abundance of “*Ca*. M. manganicus” was observed over time, starting from day 26 and ranging from 1.6 to 47.7%, with a gradual increase of “*Ca*. M. manganireducens” from 0.01% on day 26 to 23.6% on day 314 (Supplementary Table 5). The potential ability of these two species to also utilize Fe(III) is unclear, noting that both “*Ca*. M. ferrireducens” and “*Ca*. M. manganicus” were present early in the operation of the reactor when the simultaneous reduction of Mn(IV) and residual Fe(III) was detected (Fig. 1). However, given neither “*Ca*. M. manganicus” nor “*Ca*. M. manganireducens” were detected in the seed Fe(III)-fed AOM system or the original freshwater reservoir sediment [21], and both were enriched when manganese was supplied as the electron acceptor, it seems that they have a clear preference for manganese as an electron acceptor. Conversely, a substantial decrease in relative abundance of “*Ca*. M. ferrireducens,” the dominant microorganism in the parent reactor, was observed throughout the different time points starting from 23% on day 26 to 0.44% on day 405. This observation suggests that “*Ca*. M. ferrireducens” is either unable to utilize manganese, or has a lower affinity for it relative to the two “*Ca*. Methanoperedens” species that succeeded it, and does not make a substantial contribution to Mn-driven AOM in this system. In addition, MAGs belonging to “*Ca*. Methylomirabilis” (Mn-Methylomirabilis-1), known to mediate “intra-aerobic” methane oxidation [24, 53], and members of the family *Geobacteraceae* (Mn-Geobacter-1 and Mn-Geobacteraceae-1), which includes several known metal respiring species [54], were at low relative abundance (≤1.5 %) from day 152 onwards, suggesting that any contribution they made to AOM or metal reduction in the system was minimal. Analysis of 16S rRNA-based community composition profiles, generated from each metagenome using GraftM [29], showed largely congruent results with the relative abundance of the MAGs (Supplementary Table 6).

### Analysis of the anaerobic methane oxidation and energy conservation pathways in the “Ca. Methanoperedens” populations

**Fig. 4:**
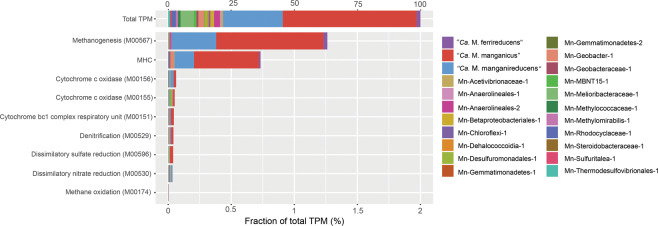
Relative expression of genes encoding microbial metabolisms of interest for the dereplicated genome set. The total transcripts per million (TPM) was calculated for each gene. KEGG annotation was used to identify ORFs coding for the methanogenesis, oxygen respiration (M00155 + M00156), dissimilatory nitrate reduction, dissimilatory sulfate reduction, and aerobic methane oxidation pathways. ORFs coding for ≥3 CXXCH motifs were used for calculating total expression levels for MHCs for each genome. Genes involved in the microbial metabolisms of interest are included in the Supplementary Dataset 1, Sheet 4—“Community members’ genes”.

In order to identify the most active microorganisms and pathways during Mn(IV)-dependent AOM, a metatranscriptome was generated from a sample collected on day 405. A large fraction of metatranscriptomic reads mapped to the MAGs recovered from the reactor (87%; Fig. 4). “*Ca*. M. manganicus” and “*Ca*. M. manganireducens” contributed 53.1% and 23.7% of total mRNA-TPM values, respectively, while analysis of the Mn-Methylomirabilis-1, Mn-Geobacter-1, Mn-Geobacteraceae-1 MAGs revealed low transcriptomic expression (Fig. 4; Supplementary Dataset 1). These results suggest that the “*Ca*. Methanoperedens” populations were the most transcriptionally active in the bioreactor at the time of sampling and collectively responsible for the bulk of the observed Mn(IV) reduction and AOM.

**Fig. 5:**
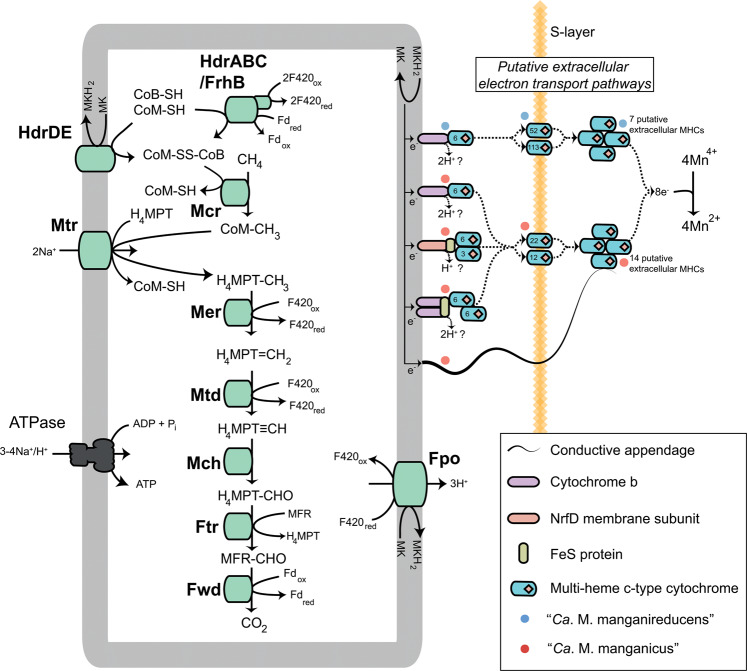
Metabolic construction of the putative pathway for AOM coupled to Mn(IV) reduction in the “*Ca*. Methanoperedens” genomes. Electrons from methane are generated through the “reverse methanogenesis” pathway and transferred into the menaquinone pool (MK/MKH_2_) via the Fpo and Hdr complexes, which oxidize F_420_H_2_ and CoM-SH + CoB-SH, respectively. Reducing equivalents are transferred via the menaquinone:cytochrome *c* oxidoreductases to MHCs located outside the cytoplasm to reduce the Mn(IV) oxides. Abbreviations for enzymes and co-factors: H_4_MPT tetrahydromethanopterin, MFR methanofuran, Fwd formylmethanofuran dehydrogenase, Ftr formylmethanofuran/H_4_MPT formyltransferase, Mch methenyl-H_4_MPT cyclohydrolase, Mtd F_420_-dependent methylene H_4_MPT dehydrogenase, Mer F_420_-dependent methylene-H_4_MPT reductase, Mtr Na^+^-translocating methyl-H_4_MPT:coenzyme M methyltransferase, Mcr methyl-coenzyme M reductase, Fpo F_420_H_2_ dehydrogenase, MK menaquinone, CoB-SH coenzyme B, CoM-SH coenzyme M, Fd ferredoxin, Hdr heterodisulfide reductase, FrhB F_420_-reducing hydrogenase subunit B, Cytb *b*-type cytochrome, NrfD polysulfide reductase subunit D, FeS ferredoxin iron–sulfur protein. The MHCs are colored blue and a number of hemes are indicated as red diamonds. Each potential MHC represented has a TPM above the median gene TPM for each species.

Analysis of the transcriptomic data from the “*Ca*. Methanoperedens” MAGs was performed to identify the genes/pathways they use to facilitate Mn(IV)-dependent AOM. Both MAGs encode and expressed a complete “reverse methanogenesis” pathway and genes encoding multiple energy converting mechanisms such as the cytoplasmic and membrane-bound heterodisulfide reductase (HdrABC and HdrDE), F_420_H_2_ dehydrogenases (Fpo), Na^+^ translocating methyltransferases (Mtr), and V-type ATPase (Fig. 5). These enzymes allow the recycling of soluble electron carriers such as coenzyme B, ferredoxin, and co-factors F_420_ that are necessary for methane oxidation, generation of a proton gradient, and production of ATP. The F_420_H_2_ dehydrogenase and the membrane-bound heterodisulfide reductase (HdrDE) are hypothesized to transfer electrons from the cytoplasm into the menaquinone pool. Genes encoding the menaquinone biosynthesis pathways (Supplementary Dataset. 1) were also identified in the “*Ca*. Methanoperedens” genomes supporting their use of menaquinone as the membrane-soluble electron carrier, consistent with other *Methanoperedenaceae* [21, 55]. The annotation of a membrane-bound formate dehydrogenase for “*Ca*. M. manganireducens” suggests the potential of this species to use formate as a carbon and electron source. Unlike the *Methanoperedenaceae* MAGs recovered from Japanese groundwater samples [51, 56], no membrane-bound H_2_ uptake NiFe hydrogenases were identified in the “*Ca*. Methanoperedens” MAGs, excluding hydrogen as an electron donor for the Mn(IV) reduction observed.

Genomic and transcriptomic analysis of the “*Ca*. Methanoperedens” MAGs reveals that electrons generated from methane oxidation are likely transferred into Mn(IV) through multiheme *c*-type cytochromes (MHCs; Fig. 5). MHCs have been previously hypothesized to mediate electron transport from ANME to syntrophic bacteria (for ANME-1 and ANME-2a,b,c [5, 8, 57]) and iron oxides (for “*Ca*. M. ferrireducens” [21]). In total, 43 and 25 putative MHCs were found to be encoded in “*Ca*. M. manganicus” and “*Ca*. M. manganireducens,” respectively (Supplementary Dataset 1). Twenty MHC protein families were found to be conserved in both “*Ca*. Methanoperedens” MAGs, five of which were co-located with menaquinone:cytochrome *c* oxidoreductase gene clusters hypothesized to transfer electrons from the menaquinone pool to MHCs outside the cytoplasmic membrane (Supplementary Dataset 1). Interestingly, the two species showed differential expression patterns in the complement of shared MHCs during AOM coupled to Mn reduction (Supplementary Dataset 1). In “*Ca*. M. manganicus,” four out of six MHC containing oxidoreductase complexes were highly expressed, with (1) one operon encoding a noncanonical bc1/b6f complex adjacent to two 6-heme MHCs, (2) an operon encoding a NrfD-like transmembrane protein, a 4Fe-4S ferredoxin iron–sulfur protein, with 3- and 6-heme MHCs, and (3) two copies of an operon encoding a *b-*type cytochrome and a 6-heme MHC (Fig. 5, Supplementary Dataset 1). In “*Ca*. M. manganireducens,” an operon encoding a *b*-type cytochrome and a 6-heme MHC was the most highly expressed of five putative menaquinone:cytochrome *c* oxidoreductase gene clusters, which is similar to the oxidoreductase gene cluster highly expressed for “*Ca*. M. ferrireducens” during Fe-driven AOM [21] (Fig. 5, Supplementary Dataset 1). These *bc* and *nrfD* complexes are frequently found in other metal reducing microorganisms as key components in electron transport from the cytoplasm to the periplasm [58–60].

In total, 23 of the 33, and 9 of the 19, MHCs predicted to be extracellular were found to be highly expressed (above the median gene TPM for the species) by “*Ca*. M. manganicus” and “*Ca*. M. manganireducens,” respectively (Supplementary Dataset 1). It has previously been reported that extracellular transfer of electrons out of the S-layer may be mediated by MHC/S-layer fusion proteins [5]. “*Ca*. M. manganicus” contained three copies of a 12 heme/S-layer protein and a 22 heme/S-layer protein, while “*Ca*. M. manganireducens” contained three MHC/S-layer proteins possessing 113, 52, and 19 hemes. All but the 19 heme MHC/S-layer protein of “*Ca*. M. manganireducens” were highly expressed indicating their importance for extracellular electron transfer (EET) for these species (Supplementary Dataset 1). This is in contrast to previous metatranscriptome analyses of “*Ca*. M. ferrireducens” and ANME-2c where MHC/S-layer proteins had been suggested to be of minor importance to EET due to their relatively low expression during AOM [21, 57]. The 113 heme MHC is the highest recorded number of hemes in a cytochrome in any microorganism, and almost double the size of the 69 heme MHC in HGW-Methanoperedenaceae-1 [56]. Several extracellular MHCs highly expressed by “*Ca*. M. manganicus” (14 MHCs) and “*Ca*. M. manganireducens (7 MHCs), that are not associated with a menaquinone reductase gene cluster and lack an S-layer protein domain, are potentially involved in facilitating the final electron transport step to birnessite (Fig. 5; Supplementary Dataset 1). Interestingly, no homologs of the extracellular MHCs that were highly expressed by “*Ca*. M. ferrireducens” during Fe-driven AOM [21] were found in the “*Ca*. M. manganicus” and “*Ca*. M. manganireducens” MAGs (Supplementary Fig. 4), suggesting these unique MHCs may be linked specifically to reduction of ferrihydrite and not birnessite. The relatively high number and diversity of the MHCs and menaquinone:cytochrome *c* oxidoreductase gene clusters, encoded by the genomes of “*Ca*. M. manganicus,” “*Ca*. M. manganireducens” and other members of the family (Supplementary Fig. 4), likely provides these microorganisms with the metabolic flexibility to utilize electron acceptors with diverse redox potentials. Further work is required to determine the function of the MHCs encoded by members of the *Methanoperedenaceae*, including their specificity for the reduction of different metal oxides (Supplementary Fig. 4).

In addition to the MHCs, conductive nanowire structures are proposed to be important for the transfer of electrons between marine ANME-1 and ANME-2c and their syntrophic sulfate reducing bacterial (SRB) partners [8, 57] These structures are suggested to allow electron transfer over greater distances relative to MHCs alone [57]. Genes encoding archaellum-like proteins were highly expressed by ANME-1a and ANME-2c in consortia with SRB were suggested to encode for such conductive structures [57] and potentially also facilitate the transfer of electrons to metal oxides. The “*Ca*. M. manganicus” and “*Ca*. M. manganireducens” both encode genes involved in the formation of archaellum, including multiple genes encoding the major subunit flagellin (*flaB*) (Fig. 5; Supplementary Dataset 1). Two of the four *flaB* genes encoded by “*Ca*. M. manganicus” were found to be highly expressed during Mn-driven AOM, while the five encoded by “*Ca*. M. manganireducens” were not expressed (Fig. 5; Supplementary Dataset 1). The high expression of these genes in “*Ca*. M. manganicus” suggest that these could be conductive appendages involved in electron transfer (Fig. 5).

Overall, this study further highlights the metabolic versatility of the *Methanoperedenaceae* lineage. Based on *meta*-omic analysis, “*Ca*. M. manganicus” and “*Ca*. manganireducens,” like the iron reducing “*Ca*. M. ferrireducens,” encode and express genes in the “reverse methanogenesis” pathway and multiple MHCs, suggesting their active role in electron transport and reduction of birnessite. Interestingly, these MHCs are differentially expressed despite their conservation in both the abundant “*Ca*. Methanoperedens” MAGs, suggesting two different mechanisms for electron transport under Mn(IV)-reducing conditions. Further investigation is required to understand the roles of these differentially expressed MHCs and their specificity for different metal oxides. Genomic characterization of the first two *Methanoperedenaceae* representatives capable of sustained Mn-driven AOM expands the known metabolic diversity of the family and provides the foundation for important future studies into their environmental relevance to the global methane and manganese cycles.

## Supplementary information

Supplemental material

Suppl. Dataset 1

Suppl. Dataset 2

## Data Availability

Sequencing data are deposited at the NCBI Sequence Read Archive under accession numbers SAMN10868419-SAMN10868422, SAMN10868423, and SAMN11109471-SAMN11109472. All draft genome nucleotide sequences have been deposited under the NCBI Biosample accession numbers SAMN10872749-SAMN10872769.
