# Supplementary material for: Anaerobic methane oxidation coupled to manganese reduction by members of the Methanoperedenaceae
Source: ISME J. 2020 Jan 27;14(4):1030–41. doi: 10.1038/s41396-020-0590-x (PMC7082337; doi:10.1038/s41396-020-0590-x)
Supplement: Supplementary file 1 — Supplemental material [file 41396_2020_590_MOESM1_ESM.pdf]

Supplementary Information:

Anaerobic methane oxidation coupled to manganese reduction by members of the *Methanoperedenaceae*

Andy O. Leu[1+], Chen Cai[2+], Simon J. McIlroy[1], Gordon Southam[3], Victoria J. Orphan[4], Zhiguo Yuan[2], Shihu Hu[2*], Gene W. Tyson[1*]

[1]Australian Centre for Ecogenomics, School of Chemistry and Molecular Biosciences, University of Queensland, Brisbane, Australia

[2]Advanced Water Management Centre, Faculty of Engineering, Architecture and Information Technology, University of Queensland, Brisbane, Australia

[3]School of Earth & Environmental Sciences, The University of Queensland, Brisbane, Queensland 4072, Australia.

[4]Department of Geological and Planetary Sciences, California Institute of Technology, Pasadena, CA 91106, USA

[+]These authors contributed equally to this work.

*Corresponding authors: Gene W. Tyson (g.tyson@uq.edu.au) and Shihu Hu (s.hu@awmc.uq.edu.au).

Table of Contents

Supplementary Figures 1-4

Supplementary Tables 1-6

Legends of Supplementary Datasets 1-2

Supplementary References

Supplementary Figures:

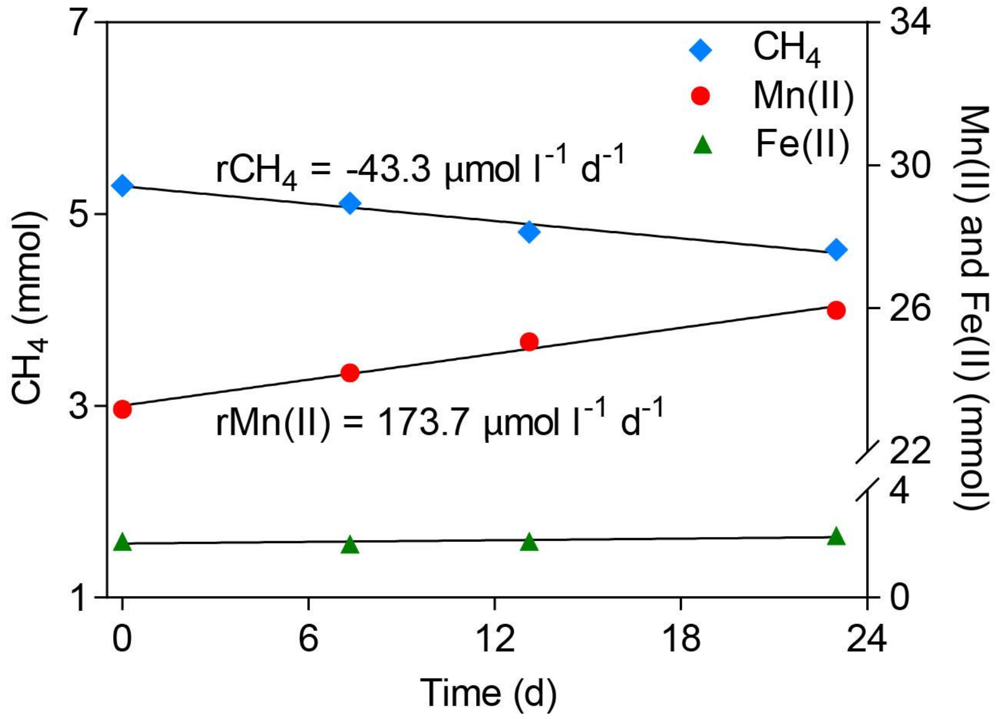

Supplementary Figure 1. Detailed bioreactor performance measurements for methane oxidation and Mn(II) reduction starting on Day 456. Each point for dissolved Mn(II)/Fe(II) represents the average of two measurements. Replication of these analyses is provided in **Figure 2**. The measured fractions of total Mn(II) are given in **Suppl. Table 1**.

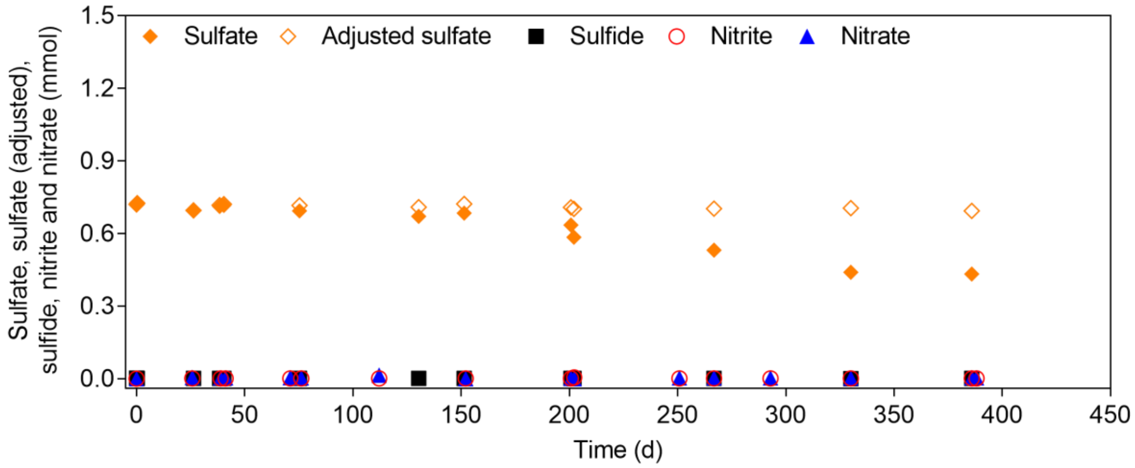

Supplementary Figure 2. Profiles of sulfate, sulfide, nitrite and nitrate during bioreactor operation. Profile of adjusted sulfate (open orange diamond) was calculated by considering the dilution factor of sampling and media replacement.

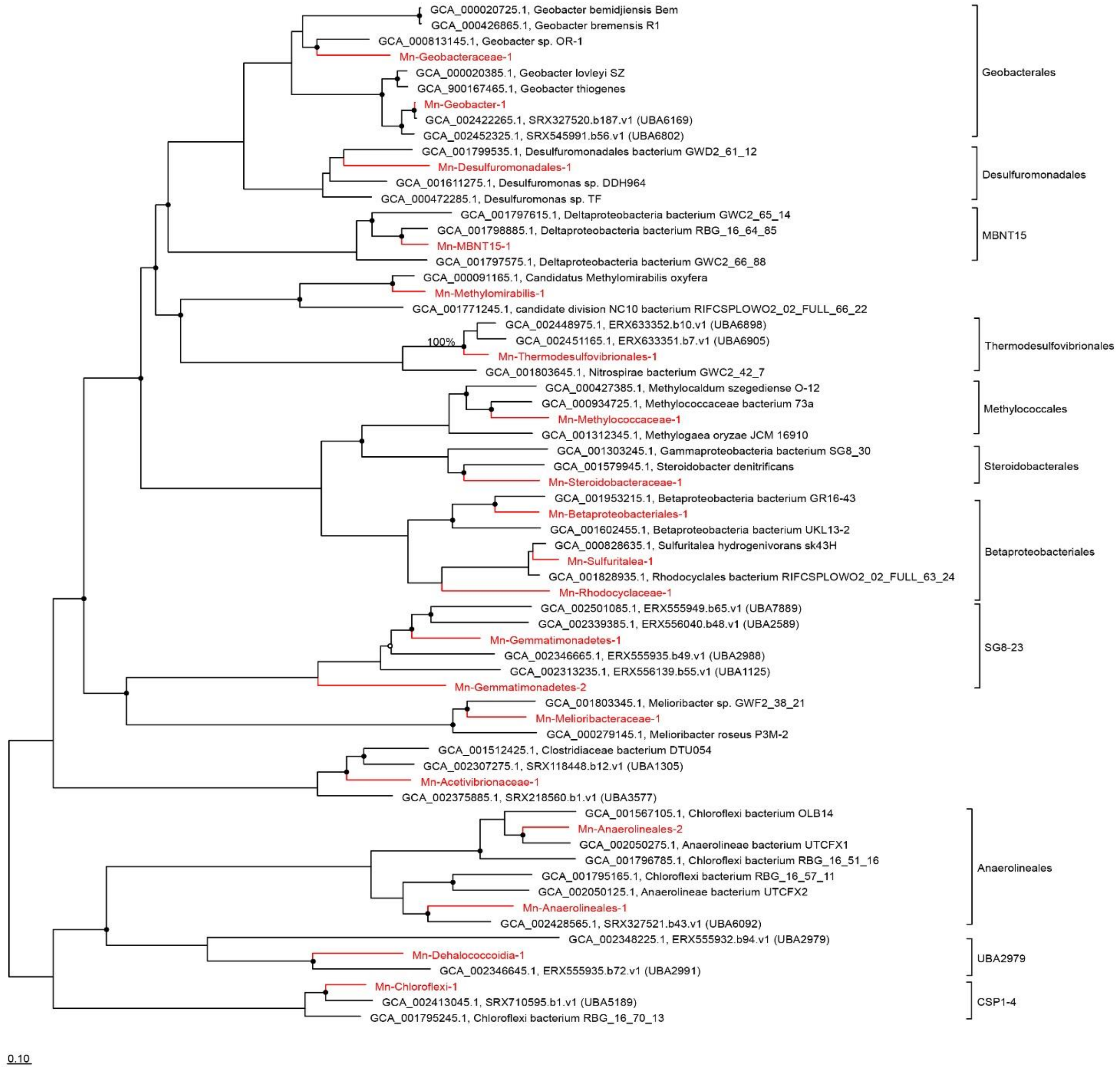

Supplementary Figure 3. Phylogenetic placement of the 19 bacterial genomes bins from this study. A maximum-likelihood tree constructed with the 19 bacterial population genomes and 36,716 bacterial reference genomes using a concatenated set of 120 bacterial-specific marker genes. Bootstrap values were determined by non-parametric bootstrapping of 100 replicates. The population genomes are highlighted in red. Black and white dots indicate ≥90% and ≥70% bootstrap support, respectively. The scale bar represents the number of amino acid substitutions per site.

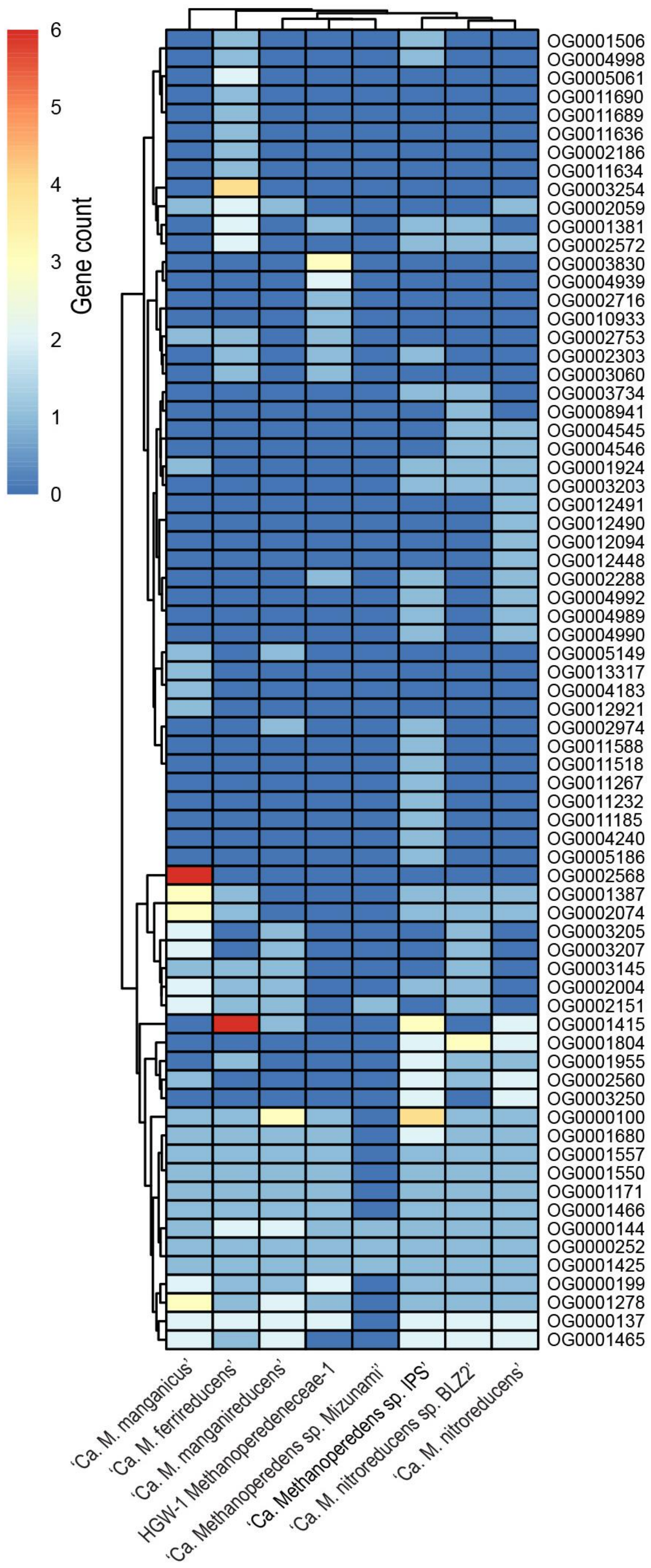

Supplementary Figure 4. Distribution of the orthologous MHC protein families across available *Methanoperedenaceae* MAGs.

Supplementary Tables:

Supplementary Table 1. Profiles for Mn(II) fractions during intensive testing periods.

Time (d)	Dissolved Mn(II) (mmol)	Adsorbed Mn(II) (mmol)	Mn(II) carbonates (mmol)
Test 1 (starting on Day 434)			
0	0.50	2.26	20.07
6.2	n.d.	2.36	21.50
16.2	n.d.	2.70	21.96
21.1	0.57	2.59	22.86
Test 2 (starting on Day 456)			
0	0.59	2.32	20.84
7.3	n.d.	2.93	21.25
13.1	n.d.	3.05	22.00
23.0	0.67	2.81	23.12

n.d.: not determined

Supplementary Table 2. Summary statistics of 21 high-quality draft genomes recovered

Bin Id	Genome size (Mbp)	No. scaffolds	N50 (scaffolds)	Strain heterogeneity#	Compl. (%)#	Cont. (%)#	GC	#CDS
‘*Ca.* M. ferrireducens’*	2.91	59	88069	0	98.69	1.31	40.8	3019
Mn-Acetivibrionaceae-1	4.21	527	10508	0	94.41	3.36	41.5	4248
Mn-Anaerolineales-1	3.53	538	8196	0	77.9	0.91	51.6	3622
Mn-Anaerolineales-2	4.51	385	16682	0	88.03	5	54.9	4643
Mn-Betaproteobacteriales-1	3.7	259	26474	25	98.39	1.22	70.5	3636
Mn-Chloroflexi-1	3.77	260	23942	0	92.87	2.78	70.7	3819
Mn-Dehalococcoidia-1	3.49	293	17122	0	89.87	3.3	66.4	3665
Mn-Desulfuromonadales-1	2.83	116	30984	0	95.16	1.45	62.2	2603
Mn-Gemmatimonadetes-1	3.57	1048	3814	0	77.27	3.3	71	3849
Mn-Gemmatimonadetes-2	3.66	481	9172	0	83.72	4.58	70.4	3468
Mn-Geobacter-1	3.52	141	35448	0	96.77	1.29	55.7	3420
Mn-Geobacteraceae-1	2.57	383	8364	20	83.1	2.58	55.4	2771
Mn-MBNT15-1	2.59	346	11982	25	96.22	2.69	65.4	2648
Mn-Melioribacteraceae-1	3.18	176	30845	0	97.77	0.56	35	2786
‘*Ca.* M. manganicus’	3.59	68	87551	0	100	1.31	40.6	3737
‘*Ca.* M. manganireducens’	3.32	116	49809	0	99.35	4.58	42.9	3684
Mn-Methylococcaceae-1	4.62	598	11714	26.32	94.82	2.13	62.7	4251
Mn-Methylomirabilis-1	2.87	249	14918	25	89.91	2.62	60.1	3046
Mn-Rhodocyclaceae-1	2.97	38	382408	0	99.53	0.71	65.7	2921
Mn-Steroidobacteraceae-1	3.14	237	19595	0	77.67	1.83	68.8	3092
Mn-Sulfuritalea-1	3.58	267	21654	22.22	85.59	3.8	62.7	3651
Mn-Thermodesulfovibrionales-1	3.04	213	22342	0	96.97	0.91	53.8	3140

All listed genomes were assessed with CheckM to be ≥70% complete with ≤5% contamination. Taxonomy was determined based on phylogenetic placement of the genomes (**Figure 3** and **Supplementary Figure 3)**. * The ‘*Ca.* M. ferrireducens’ MAG was not recovered from this study (GCA_003104905.1).

Supplementary Table 3. GTDB-Tk taxonomic classification of the dereplicated genome set.

**Genome_ID**	**GTDB-Tk taxonomic classification**
Mn-Anaerolineales-1	d__Bacteria;p__Chloroflexi;c__Anaerolineae;o__Anaerolineales;f__UBA6092
Mn-Anaerolineales-2	d__Bacteria;p__Chloroflexi;c__Anaerolineae;o__Anaerolineales;f__envOPS12;g__UBA12294;s__GCA_002418205.1
Mn-Sulfuritalea-1	d__Bacteria;p__Proteobacteria;c__Gammaproteobacteria;o__Betaproteobacteriales;f__Rhodocyclaceae;g__Sulfuritalea
Mn-Betaproteobacteriales-1	d__Bacteria;p__Proteobacteria;c__Gammaproteobacteria;o__Betaproteobacteriales;f__UKL13-2;g__GR16-43
Mn-Rhodocyclaceae-1	d__Bacteria;p__Proteobacteria;c__Gammaproteobacteria;o__Betaproteobacteriales;f__Rhodocyclaceae
Mn-Chloroflexi-1	d__Bacteria;p__Chloroflexi;c__Ellin6529;o__CSP1-4;f__CSP1-4;g__UBA5189
Mn-Dehalococcoidia-1	d__Bacteria;p__Chloroflexi;c__Dehalococcoidia;o__UBA2991;f__UBA2991
Mn-MBNT15-1	d__Bacteria;p__MBNT15;c__MBNT15;o__MBNT15;f__MBNT15;g__RBG-16-64-85
Mn-Desulfuromonadales-1	d__Bacteria;p__Desulfuromonadota;c__Desulfuromonadia;o__Desulfuromonadales;f__UBA2197
Mn-Steroidobacteraceae-1	d__Bacteria;p__Proteobacteria;c__Gammaproteobacteria;o__Steroidobacterales;f__Steroidobacteraceae
Mn-Gemmatimonadetes-1	d__Bacteria;p__Gemmatimonadetes;c__Gemmatimonadetes;o__SG8-23;f__UBA6960
Mn-Gemmatimonadetes-2	d__Bacteria;p__Gemmatimonadetes;c__Gemmatimonadetes;o__SG8-23
Mn-Geobacter-1	d__Bacteria;p__Desulfuromonadota;c__Desulfuromonadia;o__Geobacterales;f__Pelobacteraceae;g__Geobacter_C;s__GCA_002422265.1
Mn-Geobacteraceae-1	d__Bacteria;p__Desulfuromonadota;c__Desulfuromonadia;o__Geobacterales;f__Geobacteraceae
Mn-Melioribacteraceae-1	d__Bacteria;p__Bacteroidetes;c__Ignavibacteria;o__Ignavibacteriales;f__Melioribacteraceae
Mn-Methylococcaceae-1	d__Bacteria;p__Proteobacteria;c__Gammaproteobacteria;o__Methylococcales;f__Methylococcaceae;g__73a
Mn-Methylomirabilis-1	d__Bacteria;p__Methylomirabilota;c__Methylomirabilia;o__Methylomirabilales;f__Methylomirabilaceae;g__Methylomirabilis
Mn-Thermodesulfovibrionales-1	d__Bacteria;p__Nitrospirota;c__Thermodesulfovibrionia;o__Thermodesulfovibrionales;f__UBA6898;g__UBA6898
Mn-Acetivibrionaceae-1	d__Bacteria;p__Firmicutes_A;c__Clostridia;o__Acetivibrionales;f__Acetivibrionaceae

Supplementary Table 4. Average amino acid identity (AAI) of the available *Methanoperedenaceae* genomes.

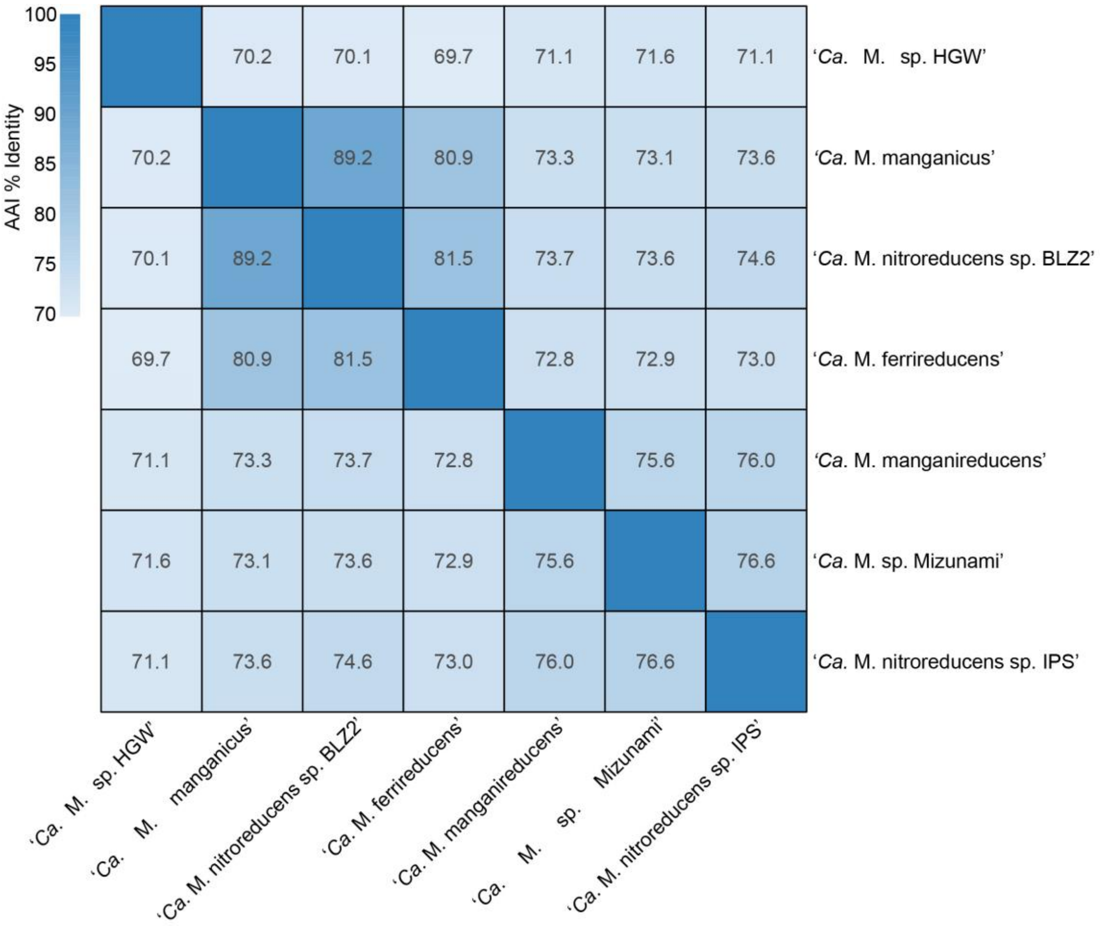

AAI were calculated using compareM. Genomes were retrieved from NCBI include: '*Ca.* M. sp. HGW' (GCA_002839545.1), '*Ca.* M. nitroreducens sp. BLZ2' (GCA_002487355.1), '*Ca.* M. ferrireducens' (GCA_003104905.1), '*Ca.* M. sp. Mizunami', '*Ca.* M. nitroreducens sp. IPS' (GCA_900196725.1), '*Ca.* M. nitroreducens' (GCA_000685155.1).

Supplementary Table 5. Relative abundance of the dereplicated genome set inclusive of '*Ca.* M. ferrireducens' (GCA_003104905.1) based on mapping of quality metagenomic reads.

**Bin Id**	**Day 26**	**Day 72**	**Day 152**	**Day 228**	**Day 314**	**Day 405**
'*Ca.* M. manganicus'	1.57	6.50	35.63	45.68	32.24	30.02
'*Ca.* M. manganireducens'	0.01	0.04	0.49	5.49	23.56	14.91
Mn-Rhodocyclaceae-1	0.03	0.09	8.11	5.92	1.43	4.89
Mn-Betaproteobacteriales-1	0.38	2.08	3.89	3.72	3.19	6.05
Mn-Melioribacteraceae-1	0.04	0.07	0.15	0.64	3.83	2.48
Mn-Methylococcaceae-1	0.02	0.33	2.34	1.25	0.56	1.05
Mn-Desulfuromonadales-1	3.94	13.55	2.60	0.76	0.38	1.02
Mn-Anaerolineales-2	0.67	1.58	1.37	0.55	0.45	1.01
'*Ca.* M. ferrireducens'	23.00	10.07	1.74	0.56	0.35	0.44
Mn-Sulfuritalea-1	0.05	2.19	1.56	0.43	0.23	0.58
Mn-Chloroflexi-1	0.13	0.23	0.33	0.32	0.64	1.46
Mn-MBNT15-1	0.17	0.45	0.81	0.60	0.44	0.84
Mn-Methylomirabilis-1	8.34	4.49	1.37	0.39	0.17	0.46
Mn-Thermodesulfovibrionales-1	1.26	1.51	1.28	0.50	0.23	0.26
Mn-Dehalococcoidia-1	0.72	0.65	0.53	0.47	0.32	0.51
Mn-Gemmatimonadetes-2	0.69	0.66	0.64	0.38	0.20	0.35
Mn-Steroidobacteraceae-1	0.03	0.06	0.18	0.33	0.28	0.58
Mn-Geobacter-1	0.00	0.00	0.14	0.15	0.33	0.65
Mn-Geobacteraceae-1	0.27	3.13	0.63	0.19	0.12	0.27
Mn-Acetivibrionaceae-1	0.64	0.36	0.31	0.26	0.29	0.25
Mn-Anaerolineales-1	0.53	0.63	0.44	0.23	0.15	0.19
Mn-Gemmatimonadetes-1	0.28	0.31	0.33	0.20	0.12	0.28
Total	42.80	49.03	64.87	69.02	69.51	68.55

The relative abundance of each population was calculated as the number of quality DNA reads mapped to the corresponding draft genome, divided by the total number of quality DNA reads.

Supplementary Table 6. Community composition based on analysis of the 16S rRNA gene fragments recruited from metagenomic reads.

**Consensus Lineage**	**Day 26**	**Day 72**	**Day 152**	**Day 228**	**Day 314**	**Day 405**
k__Archaea; p__Euryarchaeota; c__Methanomicrobia; o__Methanosarcinales; f__Methanoperedenaceae	14.8	11.4	17.7	33.7	29.6	28.9
k__Bacteria; p__Proteobacteria; c__Betaproteobacteria	1.5	3.5	9.1	7.6	6.5	7.9
k__Bacteria; p__Proteobacteria; c__Deltaproteobacteria; o__Desulfuromonadales; f__Desulfuromonadaceae	2.1	6	0	0	0	0
k__Archaea; p__Euryarchaeota; c__Methanomicrobia; o__Methanosarcinales	0	0	3.2	4.7	4.5	3.8
k__Bacteria; p__Proteobacteria; c__Betaproteobacteria; o__Ellin6067	0	0	2.4	2.8	4.4	3.6
k__Archaea; p__Euryarchaeota; c__Methanomicrobia	0	0	1.6	3.5	4.2	2.8
k__Bacteria; p__Proteobacteria; c__Betaproteobacteria; o__Rhodocyclales; f__Rhodocyclaceae	0.5	2.9	3.6	3.8	2.2	2.9
k__Bacteria; p__Chloroflexi; c__Anaerolineae	3.4	3.7	1.3	0.8	1	0.9
k__Bacteria; p__Chloroflexi; c__Anaerolineae; o__envOPS12	3.2	3.3	3.4	2.2	2.5	2.8
k__Bacteria; p__NC10; c__Methylomirabilia; g__Methylomirabilis	3.4	2.1	0	0	0	0
k__Bacteria; p__Chlorobi; c__Ignavibacteria; o__Ignavibacteriales; f__[Melioribacteraceae]	0	0	0	0.2	3	3.3
k__Bacteria; p__Parcubacteria; c__ABY1	2.9	2.7	0	0	0	0
k__Bacteria; p__Proteobacteria; c__Gammaproteobacteria; o__Methylococcales; f__Methylococcaceae; g__Methylomonas	2.9	1.6	0	0	0	0
k__Bacteria; p__Bacteroidetes; c__Bacteroidia; o__Meniscales; f__vadinHA17; g__LD21	2.8	2.3	0	0	0	0
k__Bacteria; p__Bacteroidetes; c__Bacteroidia; o__Bacteroidales	0	0	2.6	0.8	0.7	0.5
k__Bacteria; p__Proteobacteria; c__Betaproteobacteria; o__Rhodocyclales; f__Rhodocyclaceae; g__Dechloromonas	0	0	2.6	1.6	1	1.5
k__Bacteria; p__Proteobacteria; c__Deltaproteobacteria; o__Desulfuromonadales; f__Geobacteraceae; g__Geobacter	0.3	2.6	1.5	0.7	0.7	0.7

k__Bacteria; p__Chlorobi; c__Ignavibacteria; o__Ignavibacteriales	0	0	0.1	0.3	2.4	2
k__Bacteria	1.5	1.2	2.2	0.7	1.3	1.5
k__Bacteria; p__Proteobacteria; c__Deltaproteobacteria; o__MBNT15	0.4	0.8	1.6	2.2	1.7	1.7
k__Bacteria; p__Proteobacteria; c__Gammaproteobacteria; o__Xanthomonadales; f__Sinobacteraceae	0.1	0.3	1.1	1.1	2	1.8
k__Bacteria; p__Latescibacteria; c__PRR-12; o__Sediment-1	1.9	1.2	0	0	0	0
k__Bacteria; p__Chloroflexi; c__Anaerolineae; o__GCA004	0	0	1.8	1.2	1	1
k__Bacteria; p__Proteobacteria; c__Deltaproteobacteria; o__Desulfuromonadales; f__M20-Pitesti	0.2	1.8	0	0	0	0
k__Bacteria; p__Chloroflexi; c__Ellin6529	0.9	0.6	0.2	0.5	1.7	1.7
k__Bacteria; p__Proteobacteria; c__Betaproteobacteria; o__Burkholderiales	0.3	0.4	1.7	1.1	0.8	0.9
k__Bacteria; p__Aminicenantes; c__Aminicenia; o__SHA-124	1.6	1.5	0	0	0	0
k__Bacteria; p__Gemmatimonadetes; c__Gemm-1	1.6	1.3	1.2	0.6	0.4	0.3
k__Bacteria; p__Proteobacteria; c__Betaproteobacteria; o__Burkholderiales; f__Comamonadaceae	1.6	1.6	0	0	0	0
k__Archaea; p__Euryarchaeota	0	0.1	0.6	1.6	0.8	0.7
k__Bacteria; p__Nitrospirae; c__Nitrospira; o__Nitrospirales; f__[Thermodesulfovibrionaceae]; g__GOUTA19	0	0	1.6	1.1	0.9	0.4
k__Bacteria; p__Proteobacteria; c__Betaproteobacteria; o__Ellin6067; f__A0837	0.4	1.5	0	0	0	0
k__Bacteria; p__Proteobacteria; c__Betaproteobacteria; o__Burkholderiales; f__Burkholderiaceae; g__Pandoraea	1.4	0.1	0	0	0	0
k__Bacteria; p__Proteobacteria; c__Betaproteobacteria; o__Burkholderiales; f__Alcaligenaceae	0	0	0.4	0.1	0.5	1.4
k__Bacteria; p__Acidobacteria; c__Acidobacteria-6; o__iii1-15	0.9	1.3	0	0	0	0
k__Bacteria; p__Proteobacteria; c__Deltaproteobacteria	0.7	0.8	1.3	0.7	0.7	0.6

k__Bacteria; p__Proteobacteria; c__Gammaproteobacteria	0.5	0.5	1.3	1	0.9	1
k__Bacteria; p__Proteobacteria; c__Gammaproteobacteria; o__Methylococcales; f__Methylococcaceae	0.3	0.2	1.3	1.2	0.8	0.7
k__Bacteria; p__Acidobacteria; c__OS-K	1.3	1	1.1	0.3	0.3	0.2
k__Bacteria; p__Proteobacteria; c__Deltaproteobacteria; o__Myxococcales; f__Myxococcaceae; g__Anaeromyxobacter	0	0	1.2	0.4	0.3	0.4
k__Bacteria; p__Proteobacteria; c__Deltaproteobacteria; o__Myxococcales; f__Anaeromyxobacteraceae; g__Anaeromyxobacter	0.9	1.2	0	0	0	0
k__Bacteria; p__Nitrospirae; c__Nitrospira; o__Nitrospirales; f__Thermodesulfovibrionaceae; g__GOUTA19	0.8	1.2	0	0	0	0
k__Bacteria; p__Proteobacteria	0.2	0.4	1.1	0.9	0.6	0.9
k__Bacteria; p__Proteobacteria; c__Gammaproteobacteria; o__Methylococcales	1	0.3	0	0	0	0
k__Bacteria; p__Zixibacteria; c__GN04; o__V1F19b	1	1	0	0	0	0

The relative abundance abundance profiles were generated using GraftM implementing the 16S rRNA package[1].

Legends of Supplementary Datasets:

Supplementary Dataset 1. Gene annotation and TPM (Transcripts per million) values of the dereplicated genome set are listed in the "MAG annotation" sheet. TPM values of key genes involved in AOM coupled to Mn(IV) reduction for '*Ca*. M. manganicus' and '*Ca*. M. manganireducens' are listed in the "Mn-ANME-1 key metabolism" and "Mn-ANME-2 key metabolism" sheets, respectively. TPM values of selected KO genes in the KEGG pathways corresponding to Figure 4 are provided in further detail in the "Community members' gene" sheet. Overall TPM values of the KEGG pathways corresponding to Figure 4 are provided in TPM values of "KEGG modules" sheet. The MHCs annotated in the two *Methanoperedeneceae* MAGs and their predicted subcellular localisation are listed in the "MHC localisation prediction" sheet.

Supplementary Dataset 2. Archaeal-specific conserved marker genes used for phylogenetic inference are listed in the "Archaeal marker genes" sheet. Bacterial-specific conserved marker genes used for phylogenetic inference are listed in the "Bacterial marker gene" sheet.

References:

1 Boyd, J. A., Woodcroft, B. J. & Tyson, G. W. GraftM: a tool for scalable, phylogenetically informed classification of genes within metagenomes. *Nucleic acids research* (2018).
